# Editorial: Future frontiers in the management of metastatic colorectal cancer

**DOI:** 10.3389/fonc.2024.1486653

**Published:** 2024-10-04

**Authors:** Francesco Giovinazzo, Gaetano Gallo, Marta Goglia, Matteo Pavone, Alessandro Coppola, Emanuela Dell’Aquila

**Affiliations:** ^1^ Department of Surgery, Saint Camillo Hospital, Treviso, Italy; ^2^ Department of Surgery, Sapienza University of Rome, Rome, Italy; ^3^ Department of Medical and Surgical Sciences and Translational Medicine, School in Translational Medicine and Oncology, Faculty of Medicine and Psychology, Sapienza University of Rome, Rome, Italy; ^4^ Unità Operativa Complessa (UOC) Ginecologia Oncologica, Dipartimento di Scienze per la Salute della Donna e del Bambino e di Sanità Pubblica, Fondazione Policlinico Universitario A. Gemelli, IRCCS, Rome, Italy; ^5^ IHU Strasbourg, Institute of Image-Guided Surgery, Strasbourg, France; ^6^ IRCAD, Research Institute against Digestive Cancer, Strasbourg, France; ^7^ IRCCS Regina Elena National Cancer Institute, Rome, Italy

**Keywords:** colorectal cancer, metastatic colon cancer, surgery, chemotherapy, target therapy

Metastatic colorectal cancer (mCRC) remains a major challenge in oncology, with a five-year survival rate of only 14%, underscoring the need for innovative and effective treatments. Despite advances in surgery, chemotherapy, and targeted therapies, mCRC often remains incurable, highlighting the critical need for continued research and development in treatment modalities. In recent years, significant progress has been made in the treatment of mCRC, with the introduction of novel chemotherapeutic and targeted therapy agents. For instance, the combination of Trifluridine and Tipiracil with bevacizumab has emerged as a promising option for patients who have failed standard treatments. This combination has shown efficacy in prolonging survival and improving quality of life, marking a significant milestone in the management of mCRC. Indeed, in their study, Rais et al., analyzed published clinical trials of this combination, and reported that the available evidence in oncology has shown that combining bevacizumab with trifluridine/tipiracil significantly improves outcomes in heavily pre-treated metastatic mCRC patients. This combination therapy enhances progression-free survival (PFS) and overall survival (OS) compared to trifluridine/tipiracil alone, although it is associated with higher rates of severe neutropenia. Moreover, multiple studies, including the phase 3 SUNLIGHT trial, confirm these benefits, particularly in patients with RAS-mutated tumors. In fact, despite some limitations in study design, the combination has shown a good safety profile and promising efficacy.

Numerous attempts are made every day to improve our knowledge of the biological mechanisms that underlie the pathogenesis of this tumor disease with the clear aim of preventing it. To this end, Wang et al. studied the incidence of liver metastases after colorectal cancer (CRC) in the general population and the elderly population by analyzing a huge national database (with 32,330 patients) in order to identify some independent predictive factors and prognostic factors. In their analysis, they found that 13 factors have an impact on the incidence of liver metastases in the general population and these are age at diagnosis, marital status, race, bone metastases, lung metastases, CEA level, tumor size, grade, histology, primary site, T stage, N stage and sex. In the elderly population, however, 10 variables, including age at diagnosis, CEA level, tumor size, lung metastases, bone metastases, chemotherapy, surgery, N stage, grade, and race, have been shown to be independent prognostic predictors.

In addition, in their paper, Song et al. discussed the growing importance of accurately predicting lymph node metastasis in T1 CRC due to increasing early detection rates. In fact, the authors reviewed known and emerging predictors, including pathological factors, clinical tests, molecular biomarkers, and AI-based risk models, to determine the need for additional surgery after endoscopic resection. They highlighted that current predictive scoring systems for lymph node metastasis (LNM) in early CRC are mainly based on pathological parameters, but their accuracy is still limited. Some of the recent studies analyzed in their review suggest incorporating new predictors like width of submucosal invasion (WSI), area of submucosal invasion (ASI), and clinical test factors such as fibrinogen, which show greater predictive power. Molecular biomarkers and AI-based models also hold promise for improving the accuracy of LNM prediction. This could represent a major advance in the management of patients with early-stage CRC.

Surgical intervention is crucial in the management of CRC, especially when addressing liver metastases. The introduction of minimally invasive surgical techniques, such as laparoscopic and robotically-assisted liver resections, has significantly advanced the field. These techniques minimize the physical trauma of surgery, leading to shorter recovery times, reduced pain, and fewer complications compared to traditional open surgery. They maintain oncological efficacy, ensuring that cancerous tissue is effectively removed while promoting faster patient recovery and return to normal activities. Additionally, these approaches often result in better cosmetic outcomes and fewer postoperative limitations, improving overall patient satisfaction. As technology and surgical expertise continue to evolve, these minimally invasive procedures are likely to become the standard of care for CRC with liver involvement, providing patients with safer and more effective options (1).

With the purpose of comparing MIS and open technique, which has been the standard of care for many years and which is still widely used, Pinto et al. conducted a systematic review of the two different surgical approaches for surgical resection of liver metastases in CRC patients. Of the 2203 records they identified, 11 meta-analyses were selected as eligible for review, comparing MIS to open hepatectomy for CRLM. MIS consistently showed advantages, including reduced blood loss, fewer transfusions, shorter hospital stays, and lower complication rates, with similar OS and DFS compared to open surgery. However, no significant differences were found in recurrence rates or surgical margins between the two techniques, highlighting MIS as a safer alternative without compromising oncologic outcomes.

Despite significant advances, the treatment of mCRC remains burdened by challenges. A major issue is the development of ascites, as a side effect of chemotherapy, especially with drugs like Oxaliplatin. This condition can mimic symptoms of tumor recurrence, leading to potential misdiagnosis and unnecessary surgeries that pose additional risks to patients. Cui et al. reported a case report of this rare occurrence of ascites in a 65-year-old CRC patient following oxaliplatin-based chemotherapy. In their case, after ruling out tumor recurrence, the ascites resolved upon discontinuation of oxaliplatin, suggesting a possible link between the drug and ascites formation. The authors underscored the importance of distinguishing between chemotherapy-induced ascites and tumor recurrence to avoid, of course, unnecessary surgical intervention.

Furthermore, elderly patients with colorectal liver metastases face unique obstacles. They are often underrepresented in clinical trials, which leads to a lack of tailored treatment guidelines and results in suboptimal care. This demographic may experience different tolerability and responses to standard treatments, highlighting the need for more inclusive research and age-specific therapeutic strategies to ensure effective and compassionate care for all patients.

The future of mCRC treatment is poised to be transformed by advances in immunotherapy and personalized medicine. Immunotherapy is being tailored to target specific molecular and genetic alterations found in individual tumors. This precision approach promises to increase treatment efficacy while minimizing side effects. Personalized medicine, informed by genomic sequencing, enables oncologists to design bespoke treatment regimens that respond to the unique genetic landscape of a patient’s cancer.

Indeed, immunotherapy mainly targets immune checkpoints like CTLA-4 and PD-1 to enhance the immune system’s attack on tumor cells. However, the typically weak immune response in mCRC limits its effectiveness, especially in cases with proficient mismatch repair (pMMR) and microsatellite stability (MSS). While combination therapies with immunotherapy show promise, only a small subset of patients with deficient MMR (dMMR) or high microsatellite instability (MSI-H) benefit significantly (2).

The development of predictive models for lymph node metastasis also offers the potential to refine surgical strategies, ensuring that interventions are as effective and minimally invasive as possible, ultimately improving patient outcomes and survival rates.

The treatment landscape for mCRC is rapidly evolving, marked by significant advances in chemotherapy, targeted therapies, and surgical techniques. Chemotherapeutic agents are becoming more precise, minimizing side effects while maximizing efficacy. Targeted therapies focus on specific molecular pathways involved in tumor growth, offering personalized treatment options that are more effective for individual patients.

The study by Jiang et al. was presented as a systematic review and meta-analysis analyzing overall survival and progression-free survival showing that multi-target therapies and targeted therapies combined with chemotherapy demonstrate greater benefit and efficacy. Furthermore, the authors concluded that the combination of anti-VEGF drugs such as bevacizumab and aflibercept with standard chemotherapy is the treatment of choice for patients with liver metastases from CRC, although TAS-102 plus bevacizumab is superior in terms of OS according to their analysis. Indeed, their results underline the importance of translational research, with the need to guarantee targeted therapy (3).

Zheng et al. also highlighted in their detailed systematic review that targeted therapies, like those against EGFR and VEGF-R, have improved patient outcomes and quality of life, although their benefits and costs compared to chemotherapy are debated. Adoptive cell therapy is being explored for its potential, especially in patients with specific tumor antigens. The treatment landscape for mCRC continues to evolve with ongoing advances in personalized and combination therapies.

In recent years, advances in image-guided surgery (4) and artificial intelligence (5) have significantly enhanced personalized treatment by advancing the implementation of precision medicine. Intraoperative ultrasound, whether through robotic drop-in probes or laparoscopic guidance, has proven valuable in tailoring margin radicality by effectively determining the optimal transection line for rectal surgery, particularly in cases where tumors are located too high for transanal palpation (6). Additionally, in obese patients with rectal cancer, robotic intraoperative ultrasound has been shown to be beneficial in safely guiding vascular dissection (6, 7).

Surgical innovations, such as minimally invasive techniques, are shortening recovery times and improving surgical outcomes. Despite these advances, the multifaceted nature of mCRC necessitates a comprehensive approach that combines cutting-edge research, technological innovation, and personalized care strategies. This holistic approach aims to enhance survival rates and improve the quality of life for patients, emphasizing the need for continued collaboration between researchers, clinicians, and patients to overcome this complex disease.
